# Low Serum Levels of Interferon Alpha in COVID-19 Patients Are Associated with Older Age

**DOI:** 10.3390/jcm11040961

**Published:** 2022-02-12

**Authors:** Enagnon Kazali Alidjinou, Mickael Hirabidian, Anthony Rabat, Mahdi Ouafi, Magloire Pandoua Nekoua, Famara Sane, Julien Poissy, Didier Hober, The Lille COVID Research Network (LICORNE)

**Affiliations:** 1 Laboratoire de Virologie ULR3610, University of Lille, CHU Lille, F-59000 Lille, France; mickael.hirabidian@chru-lille.fr (M.H.); anthony.rabat@chru-lille.fr (A.R.); mahdi.ouafi@chru-lille.fr (M.O.); magloire-pandoua.nekoua@univ-lille.fr (M.P.N.); famara.sane@chru-lille.fr (F.S.); didier.hober@chru-lille.fr (D.H.); 2 Pôle de Réanimation, CNRS, UMR 8576-UGSF, Inserm U1285, University of Lille, CHU Lille, F-59000 Lille, France; julien.poissy@chru-lille.fr

**Keywords:** SARS-CoV-2, age, IFNα, neutralizing antibodies, severe COVID-19

## Abstract

Innate immune response, especially type 1 interferon (IFN) response is considered to play a substantial role in the outcome of SARS-CoV-2 infection. A reduced and delayed IFN response has been associated with progression to severe COVID-19. In this study, we investigated levels of circulating IFNα and serum neutralizing activity in COVID-19 patients admitted to the intensive care unit. We found a significant association of levels of IFNα with age (*p* = 0.007). This association has also been observed in a cohort of COVID-19 outpatients with mild infection (*p* = 0.02). The impact of senescence on IFN response can explain the higher susceptibility of the elderly to severe COVID-19.

## 1. Introduction

The COVID-19 pandemic has strained healthcare systems worldwide. A major challenge has been management of the overwhelming influx of severe COVID-19 patients in hospitals, and especially the intensive care units (ICU). The pathogenesis of severe COVID-19 is complex and combines factors such as hyperinflammatory responses and alterations in coagulation. Several clinical predictors of disease severity have been described such as older age, pre-existing comorbidities and hypoxia. Some markers of organ dysfunction (such as those of the heart, liver and kidneys) and non-specific markers including blood cells or coagulation abnormalities and levels of various cytokines have also been associated to severe disease [1].

Despite significant variability in the severity of clinical presentation, the patterns of antiviral response probably impact the outcome of SARS-CoV-2 infection and the related immunopathogenesis [2].

Disease severity is correlated with a dysregulated innate immune response, including a limited and delayed type I interferon (IFN) response relative to symptom onset, which probably results in high viral replication and production of an exuberant inflammatory response [3]. Several studies have revealed an impaired type 1 IFN response in severe COVID-19 patients [4,5,6].

Alterations in adaptive immunity including T cell responses and levels of neutralizing antibodies have also been described during severe COVID-19 [2].

In this study we investigated serum levels of IFNα and neutralizing antibodies, and associated factors in COVID-19 patients admitted to the ICU. In addition, serum levels of IFNα were studied in a cohort of COVID-19 mild outpatients.

## 2. Materials and Methods

### 2.1. Patients and Samples

This was a single center study conducted at the University Hospital of Lille (France). Two groups of patients were included in the study:

(i) patients admitted to the ICU for COVID-19 between March and June 2020 with a positive SARS-CoV-2 RT-PCR result using nasopharyngeal and/or lower respiratory samples and a serum sample available within 7 days upon admission and
(ii) COVID-19 outpatients with recent infection (diagnosis within 7 days after symptoms onset), and with a serum sample collected on the same day when the nasopharyngeal specimen collected and tested positive for SARS-CoV-2.

The study was conducted in the frame of the Lille COVID Research Network (LICORNE) approved by the French Institutional Authority for Personal Data Protection (Commission Nationale de l’Informatique et des Libertés DR-2020-178, 22 October 2020) and the ethics committee (ECH20/09, 7 September 2020).

### 2.2. Laboratory Methods

Various validated commercial RT-PCR assays were used for routine diagnosis of SARS-CoV-2 infection.

Anti-SARS-CoV-2 antibodies were investigated using WANTAI SARS-CoV-2 Ab enzyme-linked immunoassay (Eurobio Scientific, Les Ulis, France), which detects anti-S total antibodies.

IFNα was measured using the IFNα pan-specific ELISA kit (Mabtech^®^, Sophia Antipolis, France) that allows detection of subtypes 1/13, 2, 4, 5, 6, 7, 8, 10, 14, 16 and 17 of IFNα. The limit of detection was 7 pg/mL.

Neutralizing antibodies were investigated using a live virus neutralization assay. Briefly, serial 2-fold dilutions of the serum (starting from 1:10) were incubated at 37 °C for 1 h with 100 TCID_50_ of virus, and the mix was added to Vero E6 cell monolayers cultured in a 96-well plate. The cytopathic effect was recorded after 3 days, and the serum virus neutralization titer (VNT50) was defined as the reciprocal value of the highest dilution that showed at least 50% protection of cells. A sample with a titer ≥ 20 was defined as positive. A 20A EU2 clade SARS-CoV-2 clinical isolate (GISAID accession reference EPI_ISL_1653927) was used in all experiments. This clade was the most predominant in France during the inclusion period.

### 2.3. Statistical Analysis

GraphPad Prism version 5 (GraphPad, San Diego, CA, USA) and IBM SPSS Statistics 22 (IBM Corp., Armonk, NY, USA) softwares were used for statistical analyses. Data are presented as median and interquartile range (IQR) or as percentage. Multiple linear regression was used to identify factors associated with the levels of IFNα or neutralizing antibodies. The Mann–Whitney U test was used to compare two quantitative variables. A two-sided *p*-value < 0.05 indicated statistical significance.

## 3. Results

A total of 82 COVID-19 confirmed patients admitted to the ICU were included in the analysis. The median age was 60.5 years, and 75.6% were men. The median delay since symptoms onset was 13 days before admission, and patients were hospitalized for an average of 2 weeks in the ICU. A few patients received antiviral drugs as part of their treatment including Lopinavir/ritonavir (*n* = 14) and remdesivir (*n* = 2). Regarding the outcome 1 month (day 30) post-admission, a total of 20 deaths were recorded. Serum samples were collected 3 days (median) after the positive SARS-CoV-2 RT-PCR result. The characteristics of patients are detailed in Table 1.

Anti-SARS-CoV-2 antibodies were detected in all serum samples except in that of one patient whose sample was collected 11 days post-symptom onset.

IFNα was detectable (≥7 pg/mL) in 73 patients (89%). The median concentration was 196 (IQR: 33.2–702.4) pg/mL.

Age was significantly associated with IFNα levels (*p* = 0.007; Table 2). In addition, when patients were divided into two groups on either side of the median age, IFNα levels were found to be significantly lower in older patients than in younger patients (88.6 versus 295.4 pg/mL, *p* = 0.008).

A trend of association was found between IFNα levels and mortality at 1 month, but it did not reach statistical significance (*p* = 0.07). Similarly, the levels of IFNα were lower in patients with evolution to death at 1 month (106.5 versus 230.6 pg/mL), but the difference was not significant (*p* = 0.09).

A serum neutralizing activity was recorded in 52 patients (63.4%). The median neutralizing titer (NT50) was 40 (IQR: 20–100). The specificity of the assay was assessed using sera collected from 20 non-COVID ICU patients, and NT50 was < 20 in all samples.

Using linear regression, serum neutralizing titers were found to be associated only with body mass index (BMI) (*p* = 0.03; as shown in Table 3). However, when patients were divided into non-obese (BMI < 30) and obese (BMI ≥ 30) groups, the median neutralizing titers were similar in both groups (168.4 versus 230.6 pg/mL, *p* = 0.13).

To confirm the association between age and IFNα levels, we determined IFNα levels in 177 COVID-19 outpatients with mild infection. The serum sample and the nasopharyngeal specimen were collected on the same day. All patients were aged ≤60 years with a median age of 33 (IQR: 27–44) years. SARS-CoV-2 infection was diagnosed within the first 7 days after symptom onset (median: 2 days). The median Ct value on RT-PCR using nasopharyngeal specimens was 19.4 (IQR: 16.7–23.6).

All SARS-CoV-2 antibody test results were negative, in agreement with a recent infection.

IFNα was detected in sera from 153 patients (86.4%) with a median concentration at 98.4 (IQR: 20.8–390.6) pg/mL. As summarized in Table 4, IFNα levels were not correlated with sex, the viral load (Ct values) in nasopharyngeal specimens and the time since symptom onset (*p* = 0.88, 0.54 and 0.43, respectively). However, a significant association was found between IFNα levels and age (*p* = 0.002).

In addition, when patients were divided in two groups on either side of the median age, IFNα levels were significantly lower in the older group than in the younger group (46.5 versus 164.5 pg/mL, *p* = 0.0006).

## 4. Discussion

Immune response plays undoubtedly a major role in the pathogenesis of COVID-19. Appropriate innate immune response and especially early type I and III IFN responses can control SARS-CoV-2 replication and reduce virus-related pathogenic mechanisms [2]. Therefore, despite great interindividual variability in IFN responses, its protective role during SARS-CoV-2 acute infection is strongly suggested [3].

In this study, we investigated circulating IFNα levels and associated factors in COVID-19 patients.

IFNα was detected in most ICU patients; however, since admission to the ICU occurred several days after onset, this production might not reflect the early response. The median age in the low IFN group was significantly higher than that in the high IFN group, thus supporting reduced IFN production in older patients.

The link between advanced age and severe COVID-19 or mortality has been clearly shown [7]. The factors explaining this association probably include the impact of senescence on immune response. The mechanisms of impaired IFN production can be related to the host or virus (evasion mechanisms). Aging has been previously reported to impact IFN response during viral infections [8,9], and this can explain the higher susceptibility of the elderly to severe COVID-19 [10].

Although this association appears physiologically well known, no previous report has clearly described the correlation between age and circulating levels of IFNα. In our study, this relationship was strong because beyond ICU critical patients, it was also found in the cohort of mild COVID-19 outpatients. More interestingly, all patients in this cohort were aged ≤ 60 years, and no correlation was found with estimated viral loads, or with time since symptom onset.

Several other host factors can alter IFN production during SARS-CoV-2 infection. For example, studies have revealed that individuals with inborn errors in type I IFN-related genes are at greater risk of developing severe COVID-19 [11,12,13]. In addition, autoantibodies against type I IFN were found in 10% of patients with severe COVID-19, and not in those with mild/asymptomatic disease [14], and the prevalence of these autoantibodies was higher in older patients [15].

IFNα levels seemed to be lower in ICU patients with a poorer prognosis. The lack of strong association may be related to the limited size of our study population. However, despite the likely protective role of IFN, data are still conflicting regarding the association between IFN levels and the outcome of SARS-CoV-2 infection. A recent meta-analysis on circulating IFNα in COVID-19 patients concluded that peripheral IFNα cannot be used as a severity marker [16]. In fact, the outcome relies not only on IFNα levels but also on other factors such as the timing of production.

In this study, we also investigated serum neutralizing activity in COVID-19 ICU patients. Significant levels of neutralizing antibodies were found in more than 40% of patients, but no association with the outcome was observed. The very few data available on anti-SARS-CoV-2 neutralizing antibodies in ICU patients support that neutralizing antibody titers are strongly correlated with disease severity, because ICU patients exhibited higher nAb titers than non-ICU or outpatients with milder disease symptoms [17,18]. This observation suggests that the robust neutralizing observed in these patients does not confer protection against progression to severe COVID-19 [17].

Our study has some limitations. Firstly, the number of ICU patients was limited and might not allow to draw conclusions regarding the outcome. We also focused on IFNα levels only. The investigation of the other type I and type III IFN could provide a more complete picture of the antiviral innate response.

In conclusion, IFNα production is induced in most mild or critical COVID-19 patients, and the levels are negatively associated with age. The reduced IFNα early response may explain in part the susceptibility of older patients to more severe disease.

This finding highlights the possibility of the administration of type I IFN as a therapeutic approach in these patients. Early trials using subcutaneous injection of IFNβ reported promising results [19,20] but the recent WHO Solidarity Trial did not find an impact on mortality or hospitalization duration [21]. The effectiveness of this type of treatment and the profile of patients which might benefit from it require further studies.

## Figures and Tables

**Table 1:** Patients’ characteristics.

Characteristics	
Median age, years (IQR)	60.5 (51–70)
Sex (% of male)	75.6
Median body mass index (IQR)	29.7 (26–35)
Delay between admission and serum sample collection, days (IQR)	3 (2–5)
Delay since symptom onset, days (IQR)	13 (10–16)
Stay length in the intensive care unit, days (IQR)	14 (7–23.75)
Comorbidities, number (IQR)	1 (0–2)
Invasive ventilation (%)	72.8
Antiviral therapy (%)	19.8
Death at 1 month (%)	24.4

**Table 2:** Factors associated with IFNα levels in ICU patients (linear regression).

Characteristics	β Coefficient	*p* Value
Age	−0.363	0.007
Sex	0.089	0.47
Body mass index	0.200	0.13
Delay between admission and serum sampling	0.067	0.59
Delay since symptom onset	−0.096	0.44
Stay length in ICU	−0.153	0.30
Comorbidities	−0.074	0.56
Invasive ventilation	0.080	0.61
Antiviral therapy	−0.107	0.37
Death at 1 month	0.230	0.07

**Table 3:** Factors associated with neutralizing antibody titers in ICU patients (linear regression).

Characteristics	β Coefficient	*p* Value
Age	−0.226	0.089
Sex	0.066	0.596
Body Mass Index	0.292	0.031
Delay between admission and serum sampling	0.212	0.095
Delay since symptoms onset	0.169	0.184
Stay length in ICU	−0.01	0.948
Comorbidities	−0.059	0.641
Invasive ventilation	0.031	0.843
Antiviral therapy	−0.048	0.688
Death at 1 month	−0.038	0.761

**Table 4:** Factors associated with IFNα levels in COVID-19 outpatients (linear regression).

Characteristics	β Coefficient	*p* Value
Age	−0.24	0.002
Sex	0.011	0.88
Ct value in nasopharyngeal specimens	−0.047	0.54
Delay since symptoms onset	−0.062	0.43

## Data Availability

The data presented in this study are available on request from the corresponding author.
